# A phase 1 dose-escalation and expansion study of binimetinib (MEK162), a potent and selective oral MEK1/2 inhibitor

**DOI:** 10.1038/bjc.2017.10

**Published:** 2017-02-02

**Authors:** Johanna C Bendell, Milind Javle, Tanios S Bekaii-Saab, Richard S Finn, Zev A Wainberg, Daniel A Laheru, Colin D Weekes, Benjamin R Tan, Gazala N Khan, Mark M Zalupski, Jeffrey R Infante, Suzanne Jones, Kyriakos P Papadopoulos, Anthony W Tolcher, Renae E Chavira, Janna L Christy-Bittel, Emma Barrett, Amita Patnaik

**Affiliations:** 1 Drug Development Program, Sarah Cannon Research Institute/Tennessee Oncology, 250 25th Avenue North, Suite 200, Nashville, TN 37203, USA; 2 Department of Gastrointestinal Medical Oncology, The University of Texas MD Anderson Cancer Center, 1515 Holcombe Boulevard, Unit Number: 426, Room Number: FC10.3062, Houston, TX 77030, USA; 3 Department of Internal Medicine, Mayo Clinic, 5777 East Mayo Boulevard, Phoenix, AZ 85054, USA; 4 Department of Medicine, David Geffen School of Medicine, University of California Los Angeles, 2825 Santa Monica Boulevard, Suite 200, Santa Monica, CA 90404, USA; 5 Department of Oncology, Johns Hopkins Sidney Kimmel Comprehensive Cancer Center, 2825 Santa Monica Boulevard, Baltimore, MD 90404, USA; 6 Division of Medical Oncology, University of Colorado School of Medicine, 12801 East 17th Avenue, RC1 South, Room 8123, Aurora, CO 80045, USA; 7 Department of Medicine, Oncology Division, Washington University, School of Medicine, 14th Floor Northwest Tower, Division of Oncology, Campus Box 8056, 660 South Euclid Ave, St Louis, MO 63110, USA; 8 Department of Hematology/Oncology, Henry Ford Health System, 2799 West Grand Boulevard, Detroit, MI 48202, USA; 9 Department of Internal Medicine, University of Michigan, 1500 East Medical Center Drive, SPC 5912, Ann Arbor, MI 48109, USA; 10 Drug Development Program, Sarah Cannon Research Institute, 3322 West End Avenue, Suite 900, Nashville, TN 37203, USA; 11 Clinical Research, South Texas Accelerated Research Therapeutics (START), 4383 Medical Drive, San Antonio, TX 78229, USA; 12 Clinical Development, Array BioPharma Inc., 3200 Walnut Street, Boulder, CO 80301, USA

**Keywords:** binimetinib, MEK162, MEK inhibitor, gastrointestinal cancers, colorectal cancers, clinical trial

## Abstract

**Background:**

Binimetinib (MEK162; ARRY-438162) is a potent and selective oral MEK 1/2 inhibitor. This phase 1 study determined the maximum tolerated dose (MTD), safety, pharmacokinetic and pharmacodynamic profiles, and preliminary anti-tumour activity of binimetinib in patients with advanced solid tumours, with expansion cohorts of patients with biliary cancer or *KRAS*- or *BRAF*-mutant colorectal cancer.

**Methods:**

Binimetinib was administered twice daily. Expansion cohorts were enroled after MTD determination following a 3+3 dose-escalation design. Pharmacokinetic properties were determined from plasma samples. Tumour samples were assessed for mutations in *RAS*, *RAF*, and other relevant genes. Pharmacodynamic properties were evaluated in serum and skin punch biopsy samples.

**Results:**

Ninety-three patients received binimetinib (dose-escalation phase, 19; expansion, 74). The MTD was 60 mg twice daily, with dose-limiting adverse events (AEs) of dermatitis acneiform and chorioretinopathy. The dose for expansion patients was subsequently decreased to 45 mg twice daily because of the frequency of treatment-related ocular toxicity at the MTD. Common AEs across all dose levels included rash (81%), nausea (56%), vomiting (52%), diarrhoea (51%), peripheral oedema (46%), and fatigue (43%); most were grade 1/2. Dose-proportional increases in binimetinib exposure were observed and target inhibition was demonstrated in serum and skin punch biopsy samples. Three patients with biliary cancer had objective responses (one complete and two partial).

**Conclusions:**

Binimetinib demonstrated a manageable safety profile, target inhibition, and dose-proportional exposure. The 45 mg twice daily dose was identified as the recommended phase 2 dose. The three objective responses in biliary cancer patients are encouraging and support further evaluation in this population.

Growth factor-mediated proliferative signals are transmitted from the extracellular environment to the nucleus through several pathways, including the mitogen-activated protein kinase (MAPK) pathway (Chang *et al*, 2003b; Schulze *et al*, 2004; Roberts and Der, 2007). Activation of this pathway results in a signal cascade leading to sequential phosphorylation and activation of MAPK kinase (MEK) and extracellular signal-regulated kinase (ERK). Activated ERK regulates gene expression through phosphorylation of a variety of transcription factors that control key cellular activities, including proliferation, differentiation, migration, survival, and angiogenesis. Aberrant signalling through this pathway has been shown to lead to unconstrained cell growth and cell transformation (Scholl *et al*, 2005; Yoon and Seger, 2006), and is characteristic of many cancers.

Inappropriate MAPK pathway activation can occur through several distinct mechanisms, including activating mutations in *RAS* and *BRAF* (Bos, 1989; Davies *et al*, 2002; Lea *et al*, 2007), activated growth factor signalling (Bennasroune *et al*, 2004; Nakazawa *et al*, 2005), and cytokines and stress response signals (McCubrey *et al*, 2000; Chang *et al*, 2003a). In addition to potential cytokine involvement in tumorigenesis, increased cytokine levels may contribute to conditions such as fatigue, cachexia, and depression in patients with cancer (Reyes-Gibby *et al*, 2008). Collectively, these data suggest that targeting the MAPK pathway via MEK inhibition may inhibit cancer signalling mediated by a wide variety of signals.

Mitogen-activated protein kinase pathway activation is observed in many cancer types, including biliary and colorectal cancers (The Wellcome Trust Sanger Institute). In biliary cancer, activation appears to involve a number of events as follows: mutations in *RAS* or *BRAF* (Tannapfel *et al*, 2000; Tannapfel *et al*, 2003); aberrant activation of growth factor receptors such as ERBB family member epidermal growth factor receptor and ERBB2, and cellular mesenchymal–epithelial transition factor receptor (Aishima *et al*, 2002; Nakazawa *et al*, 2005); and stimulation of interleukin (IL)-6 (Park *et al*, 1999a; Park *et al*, 1999b). The MEK inhibitor selumetinib (AZD6244; ARRY-142886) has shown promising clinical activity as monotherapy (Bekaii-Saab *et al*, 2011), and is being tested in combination with cisplatin and gemcitabine (Bridgewater *et al*, 2016; NCT02151084), in patients with biliary cancer. Furthermore, activating mutations in *KRAS* or *BRAF* occur in approximately 50%–60% of patients with colorectal cancer; these mutations are mutually exclusive and are associated with resistance or decreased response to anti-epidermal growth factor receptor therapy in colorectal cancer (Davies *et al*, 2002; Fransen *et al*, 2004; Karapetis *et al*, 2008). Therefore, targeting MEK represents a compelling strategy for treating these diseases.

Binimetinib (MEK162; ARRY-438162) is a potent, adenosine triphosphate-uncompetitive, highly selective allosteric inhibitor of MEK1/2 with demonstrated on-target activity *in vitro* and *in vivo*, including models of cancer (Lee *et al*, 2010; Woessner *et al*, 2010). Binimetinib has nanomolar activity against purified MEK enzyme (half-maximal inhibitory concentration, 12 nM) and markedly inhibits ERK phosphorylation in human cell lines. Binimetinib potently inhibits the proliferation of a subset of cells in panels of human cancer cell lines and is particularly active in cells harbouring activating mutations in the *BRAF*, *NRAS*, and *KRAS* genes (Lee *et al*, 2010). *In vivo*, binimetinib displays broad anti-tumour activity in xenograft models derived from melanoma, colorectal cancer, non-small cell lung cancer (NSCLC), fibrosarcoma, cholangiocarcinoma, and pancreatic cancer. These non-clinical data support the use of binimetinib in a wide variety of tumour types, with a priority in tumours with aberrantly activated MAPK pathway signalling.

The primary objectives of this phase 1 study were to determine the maximum tolerated dose (MTD) of binimetinib and characterise its safety and pharmacokinetic profiles. Secondary objectives included characterisation of the pharmacodynamic profile and anti-tumour activity. Following the MTD determination, three expansion cohorts of patients with biliary cancer, *KRA*S-mutant colorectal cancer, and *BRAF*-mutant colorectal cancer were enroled to further assess the safety and clinical activity of binimetinib.

## Materials and methods

This study (NCT00959127) was conducted under all applicable regulatory requirements. The study was approved by the institutional review boards of all participating sites, and patients provided written informed consent before the initiation of study-related treatment or procedures.

### Study design and treatment

This multicentre, open-label, phase 1 study comprised two phases: a dose-escalation phase and an expansion phase. In the dose-escalation phase, a modified 3+3 design was employed to determine the MTD of binimetinib administered orally twice daily (BID) in a 21-day treatment cycles. A single dose of binimetinib was administered on day 1 and then BID continuously beginning on day 2 of cycle 1. A starting dose of 30 mg BID was utilised for the dose-escalation phase. The dose was escalated in cohorts of at least three evaluable patients at 45 mg BID, 60 mg BID, and 80 mg BID until MTD was determined. Patients in the expansion phase received continuous BID treatment with binimetinib beginning on day 1 of cycle 1 at the MTD determined in the dose-escalation phase. Patients were instructed to take BID doses 12±2 h apart with water, irrespective of food.

Patients were evaluable for dose-escalation decisions if they received at least 80% of the assigned doses or had a dose reduction, interruption, or discontinuation due to binimetinib-related toxicities during the first 21-day treatment cycle. Dose-limiting toxicities (DLTs) were any adverse event (AE) not clearly attributable to the patient's disease, including haematologic toxicities of grade 4 neutropenia for ⩾5 days, febrile neutropenia, grade 4 thrombocytopenia, and grade 3 thrombocytopenia with bleeding. Any grade 3 or 4 non-haematologic adverse events (AEs), including grade 3 nausea, vomiting, diarrhoea, aspartate aminotransferase (AST)/alanine aminotransferase (ALT) elevations >7 × the upper limit of normal (ULN) in patients with liver metastases and AST/ALT 2.5 to 5 × ULN at baseline, or rash despite maximal supportive care, were also considered dose limiting with the exception of isolated grade 3 or 4 elevations in troponin, brain natriuretic peptide (BNP), prohormone BNP, or atrial natriuretic peptide levels, unless these were associated with cardiac symptoms. Patients who required dosing interruption >21 days for drug-related AEs were classified as having experienced DLT, unless the interruption was due to grade 1/2 rash. The MTD was defined as the dose level below the dose that resulted in DLTs in ⩾33% of patients.

### Patient selection

To be eligible for any phase of this study, patients ⩾18 years of age were required to have a cardiac ejection fraction greater than or equal to the institutional lower limit of normal by echocardiogram or multigated acquisition (MUGA) scan and adequate bone marrow, renal, and hepatic function. The dose-escalation phase included patients with advanced solid tumours refractory to standard treatment, those who had no standard therapy available or chose not to pursue standard therapy, and those with an Eastern Cooperative Oncology Group (ECOG) performance status of 0 or 1, with either measurable or evaluable disease, who were willing to undergo skin punch biopsy sampling.

Patients with a history of central serous retinopathy (CSR), baseline risk factors for CSR, or retinal vein occlusion, and those who received previous MEK inhibitor treatment were excluded from the expansion phase. To be eligible for the biliary cancer expansion cohort, patients were required to have histologically or cytologically confirmed intra-hepatic or extra-hepatic cholangiocarcinoma or gallbladder carcinoma that was unresectable, locally advanced, or metastatic, and to have received no more than one prior anti-cancer therapy (including adjuvant therapy), an ECOG performance status 0 or 1, and either measurable or evaluable disease. Patients in the colorectal cancer expansion cohorts were required to have documented *KRAS*- or *BRAF*-mutant metastatic colorectal adenocarcinoma, histologically or cytologically confirmed, and to have previously received or were ineligible for 5-fluorouracil, oxaliplatin, irinotecan, and/or bevacizumab. These patients were further required to have ECOG performance status of 0 to 2 measurable disease and be willing to undergo skin punch biopsy sampling. Patients in all expansion cohorts were required to submit archival tissue or undergo a fresh biopsy for pharmacodynamic analysis.

### Safety assessments

All patients underwent a complete medical history and physical examination, assessment of ECOG performance status and vital signs, and laboratory analysis of haematology, coagulation, clinical chemistry, and urine. Three serial resting and supine 12-lead electrocardiograms were conducted over 5–10 min and cardiac ejection fraction was assessed by echocardiogram or MUGA. In addition, a complete ophthalmologic examination (including visual acuity; fundoscopy and tonometry; optical coherence tomography; and slit-lamp, lens, vitreous, and fluorescence dye examinations) was performed on each patient. These assessments were repeated throughout study participation and/or as clinically indicated. Adverse events were reviewed on an ongoing basis.

### Efficacy assessments

Efficacy was assessed through radiologic scans and clinical measurements of disease sites (if applicable), and evaluation of serologic tumour markers in patient blood samples as appropriate for tumour type. Tumour assessments were performed within 21 days before the first dose and then every 6 weeks starting at the end of cycle 2. Tumour response was evaluated by the investigator using Response Evaluation Criteria in Solid Tumours (RECIST) version 1.1 criteria. Tumour marker levels were incorporated into the overall response assessment per RECIST.

### Pharmacokinetic analysis

Blood for plasma binimetinib and metabolite concentration assessments was collected on cycle 1 day 1 (pre-dose and 30 min to 24 h post dose), cycle 1 day 15 (pre-dose and 30 min to 8 h post dose), and cycles 2 to 8 day 1 (pre-dose) from patients in the dose-escalation phase and from selected patients in the biliary cancer cohort of the expansion phase (intensive pharmacokinetic sampling). The remaining patients in the expansion phase underwent limited pharmacokinetic sampling on cycle 1 day 1 (pre-dose), cycle 1 day 8 (pre-dose), cycle 1 day 15 (pre-dose and 30 min to 8 h post dose), cycle 2 day 1 (pre-dose), cycle 3 day 1 (pre-dose and 30 min to 8 h post dose), and cycles 4–8 day 1 (pre-dose). Standard non-compartmental pharmacokinetic parameters were calculated on serial pharmacokinetic collection days (cycle 1 day 1 and cycle 1 day 15 for the dose-escalation phase; cycle 1 day 15 and cycle 3 day 1 for the expansion phase) for each patient and summarised by cohort. Dose proportionality, accumulation, and metabolite-to-parent ratio were assessed as appropriate.

### Pharmacodynamic analysis

All patients had venous blood samples collected pre-dose at baseline, cycle 1 day 1 (only if 2 samples were not collected at baseline), cycle 1 day 8, cycle 1 day 15, and day 1 of all subsequent cycles for measurement of serum tumour necrosis factor (TNF)-*α* levels. A sample was also collected at 2–4 h post dose from patients in the colorectal cancer expansion cohorts. Tumour necrosis factor-*α* was measured by a multiplexed electro-chemiluminescence assay.

Tumour samples for mutational analysis were optional in the dose-escalation phase and required in the expansion phase cohorts. Mutational analysis of *KRAS*, *NRAS*, *BRAF*, and *PI3KCa* was performed using the Sequenom OncoCarta Panel (Sequenom, San Diego, CA) and/or BEAMing digital PCR (Inostics GmbH, Hamburg, Germany) and results were reported as mutated or wild type. Phosphatase and tensin homolog (PTEN) expression was determined by immunohistochemistry using PTEN mouse monoclonal antibody (clone 6H2.1; Dako, Carpinteria, CA), visualised with 3,3′-diaminobenzidine (DAB) and counterstained with haematoxylin. Phosphatase and tensin homolog expression was reported as an H-score and classified as PTEN null (H-score<50) or PTEN positive (H-score⩾50). The absence of PTEN expression (PTEN null) indicated a *PTEN* mutation.

Skin punch biopsies (with hair follicles, if feasible) were obtained from patients in the dose-escalation phase and colorectal cancer expansion phase cohorts pre-dose at baseline and post dose within 7 days of cycle 1 day 15 for measurement of Ki67 and pERK expression. Ki67 and pERK expression were determined by immunohistochemistry using a Ki67 rabbit monoclonal antibody (clone 30-9; Ventana Medical Systems, Inc., Tucson, AZ, USA) and a pERK rabbit monoclonal antibody (Thr202/Tyr204, clone 20G11; Cell Signaling Technology, Inc., Danvers, MA), respectively, visualised with DAB and counterstained with haematoxylin. Ki67 was expressed as percentage of tumour cells with positive stain; pERK was expressed as an H-score.

### Statistical methodology

This study tested no formal hypotheses, and analyses were descriptive. The dose-escalation phase utilised a modified 3+3 design. This modified design allowed three or four evaluable patients to be enroled in a cohort, with expansion up to a total of six evaluable patients if a DLT was observed. A DLT rate of ⩾33% was considered unacceptable. It was estimated that a total of 30 patients would be treated in the dose-escalation phase. Expansion phase cohorts were planned to enrol up to 65 patients (25 patients with biliary cancer, 25 patients with *KRAS*-mutant colorectal cancer, and 15 patients with *BRAF*-mutant colorectal cancer) to further describe the tolerability at the MTD and to obtain preliminary estimates of anti-tumour activity. For an observed DLT rate of 33%, an expansion cohort of 25 patients would enable a 95% confidence interval (CI) range of 15% to 54%, and for an observed response rate of 12%, 25 patients would result in a 95% CI ranging from 3% to 31%. A Bayesian rule to monitor toxicity was employed in the expansion phase.

Adverse events and serious AEs (SAEs) were coded by preferred term (PT) and system organ class using the Medical Dictionary for Regulatory Activities version 12.0. The severity of AEs was assessed by investigators using the National Cancer Institute Common Terminology Criteria for Adverse Events version 3.0. Two AEs of special interest were captured under the composite terms of ‘combined rash' and ‘combined ocular events.' The ‘combined rash' term included the PTs of dermatitis acneiform, acne, skin exfoliation, and any term containing ‘rash'. The ‘combined ocular events' term included the PTs of retinal deposits, retinopathy, papilloedema, chorioretinopathy, macular oedema, retinal detachment, and retinal disorder.

All patients who received ⩾1 dose of binimetinib were included in the safety analysis. Patients in the safety analysis who had ⩾1 measurable lesion at baseline and at least 1 post-baseline disease assessment were evaluable for efficacy. Kaplan–Meier estimates of progression-free survival (PFS) and overall survival (OS) were performed for patients in the expansion phase (Kaplan and Meier, 1958) using SAS Version 9.2 (SAS Institute Inc., Cary, NC, USA).

## Results

Between August 2009 and May 2012, a total of 93 patients (median age, 58 years; 61% men) were enroled at 9 clinical sites in the United States. Patient characteristics are outlined in Table 1. Nineteen patients were enroled in the dose-escalation phase, with an additional 74 patients treated in the expansion phase. The expansion cohort included 28 patients with biliary cancer (60 mg BID dose group), 31 patients with *KRAS*-mutant colorectal cancer (6 patients in the 60 mg BID dose group and 25 in the 45 mg BID dose group), and 15 patients with *BRAF*-mutant colorectal cancer (45 mg BID dose group).The predominant tumour type was colorectal (57%); 54% of patients had an ECOG performance status of 0.

### Dose escalation, DLTs, and MTD

Cohorts of four patients each (three of whom were evaluable for dose-escalation decisions in each cohort) were treated at 30 and 45 mg BID without evidence of DLTs. At the 60 mg BID dose level, one patient experienced grade 2 retinopathy; although this was not considered a DLT, this cohort was expanded to six evaluable patients with no DLTs noted. Enrolment proceeded to 80 mg BID; four patients were enroled, three of whom were evaluable. The MTD was then exceeded at 80 mg BID, with two of the three evaluable patients experiencing DLTs of grade 3 chorioretinopathy and grade 3 dermatitis acneiform. Both DLTs resolved with study drug interruption and both patients resumed study drug with a dose reduction. The 60 mg BID dose was declared the MTD and was the starting dose in the expansion phase. However, after initiating the expansion phase, a higher-than-expected incidence of ocular toxicities at 60 mg BID precluded treatment of patients continuously at this dose. The starting dose was therefore reduced to 45 mg BID for newly enroled expansion phase patients.

### Safety and tolerability

Adverse events (regardless of causality) are shown in Table 2. Common AEs (all grades) included combined rash, nausea, vomiting, diarrhoea, peripheral oedema, and fatigue. Ocular events were reported in 19% of patients. Most patients experienced grade 2 (41%) or grade 3 (49%) events. The most common grade 3 events included anaemia (11%), and abdominal pain and dehydration (4% each). Grade 4 AEs were reported for 6% of patients; those that occurred in at least two patients included anaemia (3%). There were no grade 5 events. Laboratory abnormalities included increases in creatine phosphokinase (CK) and liver function tests.

Adverse events that resulted in dose reduction were reported for 16%, 49%, and 75% of patients in the 45 mg BID, 60 mg BID, and 80 mg BID dose groups, respectively. Dose reductions were most commonly due to chorioretinopathy (9%); photopsia, combined rash, and retinal detachment (3% each); and diarrhoea, fatigue, increased CK, macular oedema, myodesopsia, retinopathy, visual impairment, and vomiting (2% each).

Adverse events resulting in treatment discontinuation occurred in 16%, 15%, and 25% of patients in the 45 mg BID, 60 mg BID, and 80 mg BID dose groups, respectively. Adverse events resulting in discontinuation were most commonly due to fatigue and nausea (3% each), and combined rash and small intestinal obstruction (2% each).

Twenty-eight patients (30%) reported SAEs during the study or within 30 days of the last binimetinib dose; SAEs were reported for 50%, 20%, and 41% of patients in the 30 mg BID, 45 mg BID, and 60 mg BID dose groups, respectively. SAEs that occurred in at least two patients included anaemia (4%) and bacteraemia, dehydration, gastrointestinal haemorrhage, pneumonia, small intestinal obstruction, and ulcer haemorrhage (2% each). The maximum reported SAE severity was grade 2 for 5% of patients, grade 3 for 22%, and grade 4 for 3%. Grade 4 SAEs included anaemia and pulmonary embolism (1 patient in the 60 mg BID dose group), generalised oedema (1 patient in the 60 mg BID dose group), and cytoreductive surgery (1 patient in the 45 mg BID dose group).

Twenty patients (22%) died during the study or within 30 days of the last binimetinib dose. The cause of death for all of these patients was disease progression.

### Adverse events of special interest

Known class effects of MEK inhibitors, such as rash, ocular events, gastrointestinal events, increased CK, and oedema, were considered AEs of special interest and analysed in greater detail.

Rashes and related skin disorders were reported for 81% of patients. The incidence (77% and 88%) and median time to onset (8 days and 10 days) of combined rash were relatively similar between patients in the 45 mg BID and 60 mg BID dose groups, respectively. Combined rash was primarily grade 1 (44% of patients) or grade 2 (33% of patients), generally did not require dose modifications, and was frequently treated with concomitant medications. Three patients (3%) had grade 3 combined rash that required either dose modification (interruption and/or reduction) or treatment discontinuation.

Combined ocular events were considered related to binimetinib and were reported for 19% of patients. The incidence of ocular events was higher with increasing binimetinib dose (0%, 11%, 27%, and 50% of patients in the 30 mg BID, 45 mg BID, 60 mg BID, and 80 mg BID dose groups, respectively). Of the 18 patients with ocular events, the median time to onset after the initiation of binimetinib treatment was 17 days (range, 2–168 days). Abnormal retinal findings were observed using fundoscopy and/or optical coherence tomography. The objective findings were generally reported as macular oedema, CSR, sub-retinal fluid, or serous detachments of the retina. Ocular events were primarily grade 1 (5% of patients) or grade 2 (13% of patients) and were managed mainly with dose modifications, including brief dose interruptions and/or reductions. Ocular events were reversible in most patients upon dose reduction or discontinuation of binimetinib; 78% of patients had complete resolution, 17% were reported as recovering/resolving with retinal images documenting improvement, and 6% of patients had stable retinal findings at the time of treatment discontinuation for disease progression. One patient (1%) had a grade 3 ocular event of chorioretinopathy that was managed with a dose reduction and concomitant medications. Vascular eye events were included in the combined ocular events term; one grade 1 event of venous stasis retinopathy was reported in 1 patient (1%), which was considered related to binimetinib and led to permanent discontinuation of treatment.

Gastrointestinal events of nausea, vomiting, and diarrhoea were reported for 56%, 52%, and 51% of patients, in the 30 mg BID, 45 mg BID, and 60 mg BID dose groups, respectively. In the 45 mg BID and 60 mg BID dose groups, the incidences of nausea (61% and 51% of patients, respectively) and vomiting (52% and 49% of patients, respectively) were similar, whereas the incidence of diarrhoea was higher in the 60 mg BID dose group (61% of patients) compared with the 45 mg BID dose group (39% of patients). The maximum reported severity of nausea, vomiting, or diarrhoea was either grade 1 or grade 2. Gastrointestinal events were managed mainly with concomitant medications; however, some events required dose modifications. Three patients (3%) were permanently discontinued from treatment for nausea; none were discontinued for vomiting or diarrhoea.

Increased CK was reported for 69% of patients; 10% of patients had clinically significant CK values (i.e., values that shifted by ⩾3 grades from baseline and/or were grade 4). Abnormal CK values were reported as AEs of increased blood CK (13% of patients) and increased blood CK-MB (1% of patients), and all were considered to be related to binimetinib. Most instances of increased CK were asymptomatic. Four patients (4%) required a dose modification for AEs of increased blood CK; no patients were permanently discontinued from treatment. Most patients with elevated CK had ∼90% to 100% CK-MM.

Peripheral oedema was reported for 46% of patients. The incidences were similar in the 45 mg BID, 60 mg BID, and 80 mg BID dose groups (45%, 51%, and 50% of patients, respectively). The maximum reported severity of peripheral oedema was either grade 1 (32% of patients) or grade 2 (14% of patients) and was managed mainly with concomitant medications. One patient (1%) was permanently discontinued from treatment for two events of treatment-related grade 1 peripheral oedema.

### Pharmacokinetics

**Figure 1:**
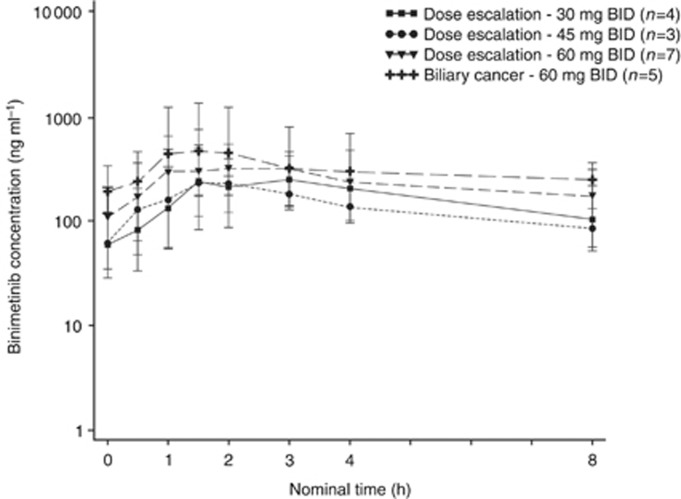
Geometric mean (s.d.) plasma binimetinib concentrations on cycle 1 day 15, intensive pharmacokinetic sampling scheme (semi-log scale).

**Figure 2:**
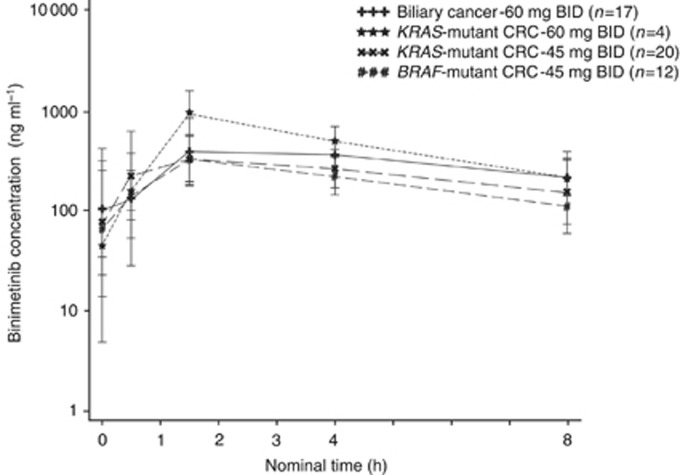
Geometric mean (s.d.) plasma binimetinib concentrations on cycle 1 day 15, limited pharmacokinetic sampling scheme (semi-log scale).

The geometric mean plasma binimetinib concentration-time profiles on cycle 1 day 15 for intensive and limited pharmacokinetic sampling are shown in Figure 1 and Figure 2, respectively. Binimetinib was approximately dose proportional over the dose range tested on days 1 and 15 of cycle 1. In the intensive pharmacokinetic sampling, the pre-dose concentrations on cycle 1 day 15 were up to threefold higher than the 12 h concentrations on day 1 and the accumulation ratios in the dose-escalation phase cohorts were 1.50 (45 mg BID cohort) and 1.17 (60 mg BID cohort), indicating moderate accumulation (Table 3). Although a formal steady-state analysis was not conducted, in the limited pharmacokinetic sampling scheme, the pre-dose concentrations were similar on cycle 1 day 15 and cycle 3 day 1 for the 45 mg BID dose group and the 60 mg BID dose group, indicating that equilibrium was reached and binimetinib was likely at steady state by cycle 1 day 15. Time of first maximum observed plasma concentration (*t*_max_) was similar, with median values of 0.5 to 4 h across all dose levels and both sampling schemes (noting the limited sampling schedule did not have samples between 1.5 and 4 h, biasing the median *t*_max_ in those cohorts). The metabolite exposure was <23% of binimetinib across dose levels.

### Pharmacodynamics and tumour tissue analysis

Pharmacodynamic blood samples for serum concentrations of TNF-*α* were collected from 78 patients. Median decreases of TNF-*α* ranging from 33% to 49% of baseline were observed at all time points across the 30 mg BID to 80 mg BID dose range, with no dose-dependent trend observed. There were no notable changes in C-reactive protein, interferon, IL-10, IL-12p70, IL-1*β*, IL-6, or IL-8.

Skin expression of Ki67, pERK, and pMEK was evaluated in pre-dose and post-dose skin punch biopsies from 33 patients. On cycle 1 day 15, median percentage of baseline levels of Ki67 and pERK ranged from 31% to 44% and 59% to 88%, respectively, across the 30 mg BID to 60 mg BID dose range; no post-dose biopsies were available from the 80 mg dose group (Table 4). There were no notable changes in pMEK relative to baseline.

Tumour tissue samples for pre-dose evaluation of mutations and PTEN expression were collected from 85 patients. Twenty patients (24%) had no mutations detected. Across all cohorts and dose levels, the most common mutations were *KRAS* (33%), *BRAF* (12%), *KRAS*+*PTEN* (7%), *KRAS*+*PI3KCA* (5%), and *PTEN* (5%). The *KRAS* mutation was most common in the *KRAS*-mutant colorectal cancer cohort, with 97% of these patients having a confirmed *KRAS* mutation. The majority of such patients had only mutations in *KRAS* (67% in 60 mg BID cohort; 72% in 45 mg BID cohort) and not in other genes analysed. Similarly, the *BRAF* mutation was most common in the *BRAF*-mutant colorectal cancer cohort, with 93% of these patients having a confirmed *BRAF* mutation and the majority of patients having only mutations in *BRAF* (60%). In the biliary cancer cohort, 72% of patients had no mutations detected. Of the 60 patients with tissue assessed for expression of PTEN, 44 patients (73%) were PTEN positive (including 16 patients in the biliary cancer cohort) and 16 patients were PTEN null.

### Response

Ninety-one patients (98%) were evaluable for response. Of these, three objective responses (3%) were reported (one complete response and two partial responses (PRs)), with durations of 11.3 months, and 10.2 and 17.9 months, respectively. All 3 of these patients had biliary cancer (3 of 30 patients with biliary cancer (10%)); 1 patient was in the 80 mg BID cohort in the dose-escalation phase, and the other 2 patients were in the biliary cancer expansion phase 60 mg BID cohort. Of the three patients who had objective responses, one tumour sample showed an *NRAS* mutation (PR patient), whereas no mutations were detected for the other two patients. An additional 33 patients (36%) had a best response of stable disease, with a median duration of 3.94 months (range, 0.92–11.53 months).

Progression-free survival and OS were estimated for patients in the expansion phase cohorts. Median PFS/OS was 1.4/7.1 months in the *BRAF*-mutant colorectal cancer cohort, 1.5/4.7 months in the *KRAS*-mutant colorectal cancer 45 mg dose cohort, 3.5/9.1 months in the *KRAS*-mutant colorectal cancer 60 mg dose cohort, and 2.1/4.8 months in the biliary cohort.

## Discussion

The dose-escalation portion of this phase 1 study determined the MTD of binimetinib to be 60 mg BID. However, because of ocular toxicities and the need for dose modifications among the initial patients treated in the expansion phase, the starting dose was reduced to 45 mg BID for the remainder of the expansion phase and is the recommended phase 2 dose for subsequent single-agent clinical studies. The 45 mg BID dose was also identified as the MTD/recommended phase 2 dose in a recent phase I study of binimetinib monotherapy conducted in Japan in patients with advanced solid tumours (Watanabe *et al*, 2016). Consistent with the known class effects of MEK inhibition (Adjei *et al*, 2008; Rosen *et al*, 2011; Infante *et al*, 2012), common AEs included rash, diarrhoea, nausea, peripheral oedema, vomiting, fatigue, and ocular events. Laboratory abnormalities included increases in CK and liver function tests. The pharmacokinetic profile of binimetinib was approximately dose proportional over the range of doses evaluated. Pharmacodynamic studies demonstrated target inhibition, with decreases in TNF-*α* observed in serum samples and decreases in Ki67 and pERK levels observed in skin punch biopsy samples.

A number of MEK inhibitors have been evaluated in clinical trials; to date, trametinib, cobimetinib, and binimetinib are the only agents in this class to demonstrate efficacy in phase 3 trials of melanoma (Flaherty *et al*, 2012; Larkin *et al*, 2014; Long *et al*, 2014; Robert *et al*, 2015; Dummer *et al*, 2016). Trametinib is indicated in the United States as a single agent and in combination with the BRAF inhibitor dabrafenib for *BRAF*V600E- or *BRAF*V600K-mutant metastatic melanoma (MEKINIST, 2014). Cobimetinib is indicated in the United States for unresectable or metastatic melanoma with a *BRAF*V600E or *BRAF*V600K mutation, in combination with vemurafenib (Lee *et al*, 2015). Binimetinib showed clinical activity in a phase 2 study (NCT01320085), yielding a 15% response rate in patients with advanced *NRAS-*mutant melanoma (Ascierto *et al*, 2013; Van Herpen *et al*, 2015). In the subsequent phase 3 NEMO study (NCT01763164), binimetinib met its primary endpoint, conferring significantly longer PFS *vs* dacarbazine in patients with *NRAS*-mutant melanoma (Dummer *et al*, 2016).

In tumour types other than melanoma, responses to MEK inhibition have been less common. In the first-in-human trial of trametinib, 21 patients had objective responses, only 4 of whom were non-melanoma patients (NSCLC and pancreas cancer (2 patients each) NCT00687622) (Infante *et al*, 2012). In addition, no objective responses to single-agent treatment were reported in non-melanoma patients in phase 1 trials of the MEK inhibitors selumetinib (NCT00085787) (Adjei *et al*, 2008), cobimetinib (NCT00467779) (Rosen *et al*, 2011), or pimasertib (NCT00982865) (Delord *et al*, 2010).

In the current trial, three patients with biliary cancer had an objective response to binimetinib treatment. However, of these three patients, two had no mutations identified and 1 had an *NRAS* mutation, suggesting no correlation between mutation status and objective response in this study. This is consistent with data reported from a phase 2 trial of selumetinib in patients with metastatic biliary cancer, in which three patients (12%) achieved an objective response, none of whom had mutations in *RAF* or *RAS* (Bekaii-Saab *et al*, 2011). Disappointingly, no responses to binimetinib were observed in patients with colorectal cancer in either the *KRAS*- or *BRAF*-mutant cohorts; this result is consistent with other single-agent clinical studies of MEK inhibitors in patients with colorectal cancer (Rinehart *et al*, 2004; Zimmer *et al*, 2014) and suggests combination therapy may be needed to treat this tumour type.

In summary, binimetinib was safe and tolerable at 45 mg BID, with preliminary anti-tumour activity demonstrated in patients with biliary cancer. Additional characterisation of the response to binimetinib in the biliary cancer expansion cohort in this study is underway (manuscript in preparation) and further evaluation of binimetinib in combination with gemcitabine and cisplatin in a phase 1 study (NCT01828034) is ongoing. A preliminary report noted encouraging results, with six patients experiencing partial responses and four experiencing stable disease among the 12 patients who participated in the study (Lowery *et al*, 2015). Binimetinib is also undergoing evaluation as monotherapy and in combination with targeted and cytotoxic chemotherapies in other tumour types known to have MAPK pathway activation, including melanoma, NSCLC, and pancreatic, colorectal, and thyroid cancers. Combining binimetinib with other therapies is a promising strategy to overcome or delay resistance that has been observed with MEK inhibition. A phase 3 trial of binimetinib in combination with the BRAF inhibitor encorafenib (LGX818) for *BRAF*V600E-mutant metastatic melanoma is also ongoing (NCT01909453).

## Figures and Tables

**Table 1:** Patient characteristics

**Characteristic**	***N*****=93**
Median age, years (range)	58 (30–86)
**Sex, *n* (%)**	
Men	57 (61)
Women	36 (39)
**Race, *n* (%)**	
White	80 (86)
Black/African American	7 (8)
Asian	3 (3)
Other	3 (3)
**Tumour type, *n* (%)**	
Colorectal	53 (57)
Biliary	30 (32)
Pancreas	3 (3)
Other^a^	7 (8)
**ECOG performance status, *n* (%)**	
0	50 (54)
1	42 (45)
2	1 (1)
Median prior regimens for advanced/metastatic disease (range)	2 (0–10)
**Prior treatments,^b^ *n* (%)**	
Radiation	30 (32)
Surgery	73 (78)
Adjuvant therapy	48 (52)
**Enrolment, *n* (%)**	
Dose-escalation phase	19 (20)
30 mg BID	4 (4)
45 mg BID	4 (4)
60 mg BID	7 (8)
80 mg BID	4 (4)
Expansion phase^c^	74 (80)
Biliary cancer cohort (60 mg BID)	28 (30)
*KRAS*-mutant colorectal cancer cohort (60 mg BID)	6 (6)
*KRAS*-mutant colorectal cancer cohort (45 mg BID)	25 (27)
*BRAF*-mutant colorectal cancer cohort (45 mg BID)	15 (16)

Abbreviations: BID=twice daily; ECOG=Eastern Cooperative Oncology Group; MTD=maximum tolerated dose.

a Includes appendiceal, carcinoma of unknown primary, eccrine adenocarcinoma, gastric, melanoma, nerve sheath tumour, and parotid.

b Patients may have received more than 1 prior cancer treatment.

c After initiating the expansion phase, a higher-than-expected incidence of ocular AEs affected the ability to treat patients continuously at the MTD of 60 mg BID. The dose was therefore reduced to 45 mg BID for the remainder of newly enroled expansion phase patients.

**Table 2:** Adverse events, regardless of causality, reported in ⩾15% of patients, all grades by dose level (*N*=93)

	**30 mg BID (*****n*****=4)**		**45 mg BID (*****n*****=44)**		**60 mg BID (*****n*****=41)**		**80 mg BID (*****n*****=4)**		
**Adverse event,** ***n*** **(%)**	**Gr 1/2**	**Gr 3/4**	**Gr 1/2**	**Gr 3/4**	**Gr 1/2**	**Gr 3/4**	**Gr 1/2**	**Gr 3/4**	**Total (*****N*****=93)**
Combined rash^a^	4 (100)	0 (0)	33 (75)	1 (2)	35 (85)	1 (2)	0 (0)	1 (25)	75 (81)
Nausea	3 (75)	0 (0)	27 (61)	0 (0)	21 (51)	0 (0)	1 (25)	0 (0)	52 (56)
Vomiting	3 (75)	0 (0)	23 (52)	0 (0)	20 (49)	0 (0)	2 (50)	0 (0)	48 (52)
Diarrhoea	4 (100)	0 (0)	17 (39)	0 (0)	25 (61)	0 (0)	1 (25)	0 (0)	47 (51)
Peripheral oedema	0 (0)	0 (0)	20 (45)	0 (0)	21 (51)	0 (0)	2 (50)	0 (0)	43 (46)
Fatigue	2 (50)	0 (0)	18 (41)	2 (5)	16 (39)	1 (2)	1 (25)	0 (0)	40 (43)
Anaemia	1 (25)	0 (0)	5 (11)	3 (7)	5 (12)	10 (24)	0 (0)	0 (0)	24 (26)
Abdominal pain	1 (25)	0 (0)	7 (16)	1 (2)	7 (17)	3 (7)	1 (25)	0 (0)	20 (22)
Anorexia	1 (25)	0 (0)	8 (18)	0 (0)	8 (20)	0 (0)	1 (25)	0 (0)	18 (19)
Combined ocular events^b^	0 (0)	0 (0)	5 (11)	0 (0)	11 (27)	0 (0)	1 (25)	1 (25)	18 (19)
Constipation	2 (50)	0 (0)	6 (14)	0 (0)	8 (20)	2 (5)	0 (0)	0 (0)	18 (19)
Dyspnoea	2 (50)	0 (0)	5 (11)	2 (5)	6 (15)	1 (2)	1 (25)	0 (0)	17 (18)
Pyrexia	2 (50)	0 (0)	7 (16)	0 (0)	7 (17)	1 (2)	0 (0)	0 (0)	17 (18)
Dizziness	0 (0)	0 (0)	5 (11)	0 (0)	9 (22)	0 (0)	0 (0)	0 (0)	14 (15)

Abbreviations: BID=twice daily; Gr=grade; PT=preferred term.

a Combined rash term includes PTs of dermatitis acneiform, acne, skin exfoliation, and any term containing rash.

b Combined ocular events term includes PTs of retinal deposits, retinopathy, papilloedema, chorioretinopathy, macular oedema, retinal detachment, and retinal disorder.

**Table 3:** Plasma pharmacokinetic parameters for cycle 1 day 1 and cycle 1 day 15 for the intensive pharmacokinetic sampling scheme

			**Dose-escalation phase**				**Expansion phase**	
**Cycle**	**Day**	**Parameter**	**30 mg BID (*****n*****=4)**	**45 mg BID (*****n*****=4)**	**60 mg BID (*****n*****=7)**	**80 mg BID (*****n*****=4)**	**Biliary 60 mg BID (*****n*****=7)**	**Total (*****N*****=26)**
1	1	AUC_0–8_, h*ng ml^−1^^a^	1000 (34.0)	964 (28.4)	1710 (23.9)	2220 (78.9)	1090 (293)	NR
		*C*_max_, ng ml^−1^^a^	327 (28.6)	241 (43.2)	545 (32.3)	687 (66.6)	365 (141)	NR
		*t*_max_, h^b^	1.51 (1.00–4.07)	2.53 (1.50–3.02)	1.00 (0.500–4.08)	2.02 (1.00–10.0)	1.50 (1.13–10.0)	1.50 (0.500–10.0)
1	15	AUC_0-8_, h*ng ml^−1^^a^	NA (NA)	1490 (NC)	1820 (14.4)	NA (NA)	3760 (NC)	NR
		*C*_max_, ng ml^−1^^a^	417 (39.9)	273 (64.7)	512 (30.8)	NA (NA)	594 (68.8)	NR
		*R*_AUC_^a^	NA (NA)	1.50 (NC)	1.17 (18.4)	NA (NA)	2.50 (NC)	1.44 (32.8)
		*t*_max_, h^b^	1.50 (1.50–3.83)	2.00 (1.07–2.87)	3.00 (0.533–7.12)	NA (NA)	1.50 (1.00–7.02)	1.50 (0.533–7.12)

Abbreviations: AUC=area under the plasma concentration-time curve; AUC_0–8_=AUC from time 0 to 8 h; BID = twice daily; *C*_max_=first maximum observed plasma concentration; CV=coefficient of variation; NA=not applicable; NC=not calculated; NR=not reportable; *R*_AUC_=accumulation ratios.

a Geometric mean (% CV).

b Median (minimum–maximum).

**Table 4:** Skin expression of Ki67 and pERK at cycle 1 day 15

**Median (range) percent of baseline**	**30 mg BID (*****n*****=4)**	**45 mg BID (*****n*****=22)**	**60 mg BID (*****n*****=7)**	**Total (*****N*****=33)**
Ki67	44 (25–56)	33 (6–300)	31 (17–100)	33 (6–300)
pERK	88 (50–100)	66 (38–189)	59 (29–72)	64 (29–189)

Abbreviations: BID=twice daily.
